# Chitosan and Cyclodextrins—Versatile Materials Used to Create Drug Delivery Systems for Gastrointestinal Cancers

**DOI:** 10.3390/pharmaceutics16010043

**Published:** 2023-12-27

**Authors:** Alfred Najm, Adelina-Gabriela Niculescu, Alexandra Bolocan, Marius Rădulescu, Alexandru Mihai Grumezescu, Mircea Beuran, Bogdan Severus Gaspar

**Affiliations:** 1 Department of Surgery, Carol Davila University of Medicine and Pharmacy, 8 Eroii Sanitari, Sector 5, 050474 Bucharest, Romania; alfred.najm@yahoo.ro (A.N.); drmirceabeuran@yahoo.com (M.B.); bogdangaspar2005@yahoo.com (B.S.G.); 2 Emergency Hospital Floreasca Bucharest, 8 Calea Floresca, Sector 1, 014461 Bucharest, Romania; 3 Research Institute of the University of Bucharest—ICUB, University of Bucharest, 050657 Bucharest, Romania; adelina.niculescu@upb.ro (A.-G.N.); grumezescu@yahoo.com (A.M.G.); 4 Department of Science and Engineering of Oxide Materials and Nanomaterials, Politehnica University of Bucharest, 011061 Bucharest, Romania; 5 General Surgery Department, Carol Davila University of Medicine and Pharmacy, The University Emergency Hospital of Bucharest, 050098 Bucharest, Romania; bolocan.alexa@gmail.com; 6 Department of Inorganic Chemistry, Physical Chemistry and Electrochemistry, Politehnica University of Bucharest, 011061 Bucharest, Romania; 7 Academy of Romanian Scientists, Ilfov No. 3, 050044 Bucharest, Romania

**Keywords:** gastrointestinal cancers, drug delivery systems, chitosan-based nanocarriers, cyclodextrin-based delivery systems, inclusion complexes, novel antitumor approaches

## Abstract

Gastrointestinal cancers are characterized by a frequent incidence, a high number of associated deaths, and a tremendous burden on the medical system and patients worldwide. As conventional chemotherapeutic drugs face numerous limitations, researchers started to investigate better alternatives for extending drug efficacy and limiting adverse effects. A remarkably increasing interest has been addressed to chitosan and cyclodextrins, two highly versatile natural carbohydrate materials endowed with unique physicochemical properties. In this respect, numerous studies reported on fabricating various chitosan and cyclodextrin-based formulations that enabled prolonged circulation times, improved cellular internalization of carried drugs, preferential uptake by the targeted cells, reduced side effects, enhanced apoptosis rates, and increased tumor suppression rates. Therefore, this paper aims to briefly present the advantageous properties of these oligo- and polysaccharides for designing drug delivery systems, further focusing the discussion on nanocarrier systems based on chitosan/cyclodextrins for treating different gastrointestinal cancers. Specifically, there are reviewed studies describing promising solutions for colorectal, liver, gastric, pancreatic, and other types of cancers of the digestive system towards creating an updated framework of what concerns anticancer chitosan/cyclodextrin-based drug delivery systems.

## 1. Introduction

Cancer is a prominent group of fatal diseases that pose tremendous challenges to the healthcare systems and affect millions of patients worldwide [1,2]. Globally, gastrointestinal cancers are among the most commonly occurring types of cancers, accounting for more than 25% of the total cancer incidence cases and more than 33% of cancer-related deaths [3,4,5].

Chemotherapeutic, radiotherapeutic, and surgical approaches have been used to treat gastrointestinal cancers, leading to various degrees of success, yet they have also been associated with numerous negative aspects. Specifically, these conventional strategies exhibit low cancer inhibition efficiency, non-specific drug delivery, and severe adverse effects [6]. Moreover, classic chemotherapeutic drugs have reduced water solubility, lack stability, allow only a short drug cycle time, exhibit poor bioavailability, require high doses for reaching the tumor site in an optimum concentration, and produce systemic side effects [5,7,8].

Thus, it is essential to improve drug behaviors by loading them into specialized carriers. In this respect, a plethora of nanomaterials have been researched for fabricating biocompatible delivery systems for anticancer drugs [5,7]. Including chemotherapeutics in engineered nanovehicles is promising for reducing unwanted cytotoxicity, improving solubility and stability, and ensuring controlled and sustained drug release [9,10].

One attractive alternative to designing carriers for anticancer medicines implies using cyclodextrins. These cyclic oligosaccharides of natural origin are recognized for their safe profile, ability to encapsulate various moieties within their cavity, and the possibility of facile chemical modification [1,5]. An equally appealing strategy supposes the use of chitosan for developing state-of-the-art cancer delivery systems. Specifically, this natural polymer benefits from biocompatibility, biodegradability, good serum stability, long-term circulation time, and mucodhesiveness [6,11,12,13].

Therefore, given their favorable physicochemical properties, chitosan and cyclodextrins have drawn increasing attention for elaborating better-performing anticancer therapeutics. In this context, this paper takes a comprehensive path in discussing chitosan/cyclodextrin-based drug delivery systems for gastrointestinal cancers, starting by describing the characteristics of interest of these saccharides for this particular type of delivery, and further moving to their applicability in colorectal, liver, gastric, pancreatic and other types of cancers of the digestive system.

## 2. Properties of Interest of the Carbohydrate Materials for Drug Delivery

### 2.1. Chitosan

**Figure 1:**
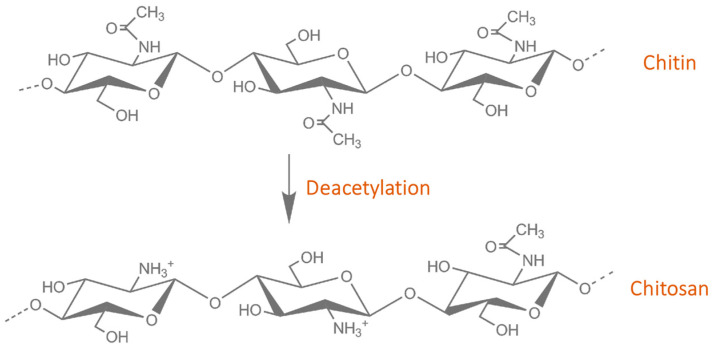
Molecular structures of chitin and chitosan. Adapted from an open-access source [21].

Chitosan represents a safe, natural polymer obtained from chitin (Figure 1), a highly abundant polysaccharide found in marine crustaceans [14,15,16,17]. Chitosan has been rendered promising in numerous applications, including food technology and medical and pharmaceutical uses [18,19]. Safe for human consumption, chitosan has been used as a food additive in several countries, approved for biomedical devices, such as hemostatic dressings and bandages, and as a coating agent for contact lenses [20]. Moreover, its appealing biological properties recommend this natural polymer for tissue engineering, gene delivery, and drug-carrying systems [16,17].

Chitosan is recognized for its nontoxicity, biocompatibility, biodegradability, hemocompatibility, wound healing efficiency, homeostasis, and exudate absorption capacity. This polymer also exhibits attractive biological activities, including antitumor, antioxidant, and antimicrobial effects [22,23,24,25]. Furthermore, it can be combined or conjugated with other polymers (e.g., alginate, polylysine, poly(γ-glutamic acid), and short-chain amylose), providing materials with improved characteristics [17,24].

In addition, chitosan possesses mucoadhesive properties and an abundance of modifiable functional groups (i.e., hydroxyl, amine, and carboxyl groups) that facilitate trans-mucosal drug delivery [12,26]. Thus, after being adsorbed to the mucous membrane along the gastrointestinal tract, chitosan holds promise as a carrier for different agents that cannot be otherwise delivered to certain areas of the digestive system [17,27]. In particular, given its antitumor potential, this polysaccharide can be employed for carrying and releasing chemotherapeutic drugs to gastrointestinal cancer-affected regions [28].

The anticancer activity of chitosan has been correlated with its cationic nature, molecular weight, and deacetylation degree, with negligible toxicity to healthy cells [20]. Structurally, this natural polymer contains randomly distributed β-(1→4)-linked deacetylated and N-acetylated units of D-glucosamine (Figure 1). Depending on the source and preparation methods, chitosan can have a molecular weight in the range of 5 to 1000 kDa and a deacetylation degree (DD) between 30 and 95% [29,30]. It has been reported that an increase in the DD is correlated with an increase in the chitosan charge density, leading to improved drug transportation and enhanced epithelial permeability [30]. From the polymerization degree point of view, low molecular weight (LMW, ∼5 kDa) polysaccharide interactions were observed to be influenced by the intrinsic charge of chitosan, while for high molecular weight (HMW, ∼135 kDa) polymer interactions, the main influencing element was chain flexibility [31].

Furthermore, this polymer has been noted to undergo protonation and solubilization to a greater extent in acidic environments, which are characteristic of tumoral tissues. Moreover, through its high zeta potential, chitosan can effectively interact with the negatively charged membranes of cancer cells and endothelial cells of the tumor vasculature, altering cell membrane permeability, entering the cell, and exerting its anticancer effects by suppressing matrix metallopeptidase 9 (MMP 9) protein production [20]. Additionally, this polymer reportedly interferes with cell metabolism, inhibiting cell growth or inducing cell apoptosis [32].

In its nanoparticulate form, chitosan allows the slow/controlled release of carried agents, improving drug solubility, stability, and efficacy [32,33]. Through their reduced dimensions and ability to transiently open the tight junctions between epithelial cells, chitosan nanocarriers can safely and effectively deliver encapsulated drugs, increasing their retention time and enhancing the cellular uptake probability of hydrophilic therapeutic agents [16].

Chitosan exhibits a plethora of beneficial functional characteristics; however, its application in drug delivery is limited by a few issues. This carbohydrate has a high hydrophilicity and swelling degree but reduced thermal stability and ductility. Its poor solubility at physiological pH (pH 7.4) is often considered a limitation in drug delivery applications, as chitosan is generally transformed to its chemical derivatives through acylation, carboxymethylation, quaternization, and thiolation reactions [29,30]. However, in the context of anticancer applications, the insolubility of this natural polysaccharide can be considered an advantage, enabling drug release only at the affected location characterized by an acidic pH.

It has also been reported that as chitosan-based carriers pass through the intestines, their features gradually alter, especially their DD. Given that DD can further impact carrier characteristics, including the swelling ratio, mucoadhesion, and drug release profile, all of which have a significant impact on the delivery system’s performance, it is advised to take these factors into account when developing chemotherapeutic carriers aimed at short-term biodegradation applications [34].

### 2.2. Cyclodextrins

**Figure 2:**
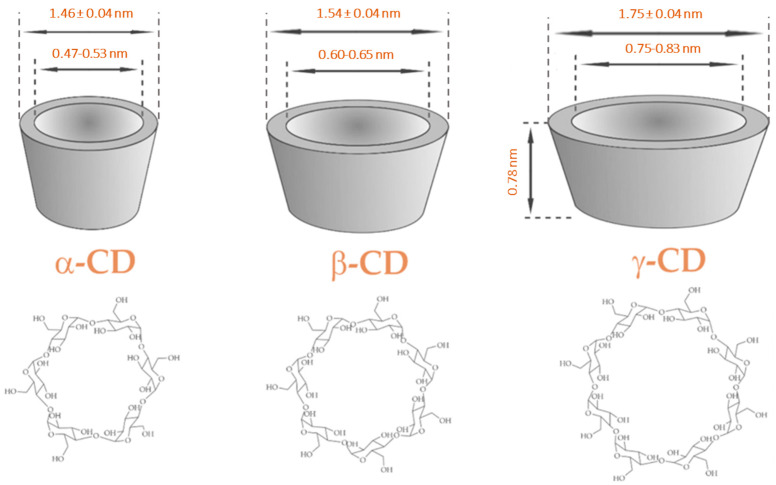
α-, β-, and γ-CD (cyclodextrin) molecules. Created based on information from [5,43,44,45,46].

Cyclodextrins are natural cyclic oligosaccharides obtained from the enzymatic hydrolysis of starch. Natural cyclodextrins comprise six, seven, or eight glucose units linked by α-1,4 glycosidic bonds, being called α, β, and γ-cyclodextrin, respectively [35,36,37] (Figure 2). These chemical compounds present a hollow truncated cone morphology with an external hydrophilic surface and internal hydrophobic cavity [37,38,39,40,41]. Given their convenient structure and ease of chemical modification, cyclodextrins represent versatile excipients for drug delivery [38,40,42].

The structural arrangement of cyclodextrins permits the inclusion of hydrophobic drugs into their cavity, generating host–guest complexes by noncovalent interactions, which are of great use in pharmaceutical applications [40]. The formation of inclusion complexes does not necessitate complicated chemical reactions, which are mediated by intermolecular forces, such as hydrogen bonding, van der Waals forces, hydrophobic interactions, and ionic interactions, depending on whether or not the drug is charged [47,48].

**Figure 3:**
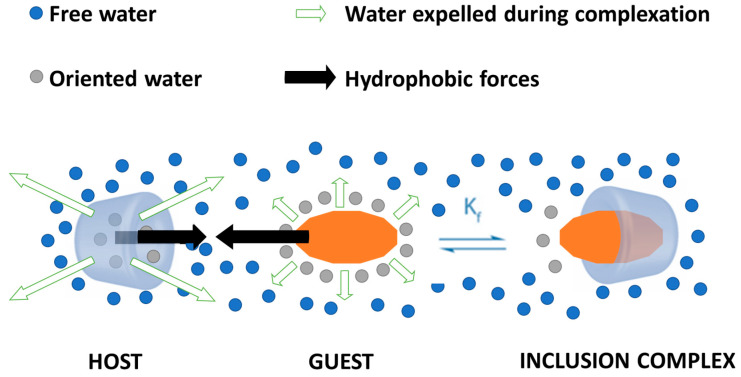
Schematic representation of the formation of an inclusion complex between CD (host) and a guest. Reprinted from an open-access source [5].

Such inclusion complexes can improve the apparent water solubility of the drugs and enhance their stability for reaching the target area in an appropriate concentration while also reducing unwanted side effects [40,49]. Furthermore, it is possible to obtain reversible host–guest complexes between cyclodextrins and different moieties, thus allowing not only efficient encapsulation but also cargo release at the desired site [50,51,52]. In more detail, inclusion complexes are formed under a dynamic association/dissociation equilibrium between free guest molecules, un-complexed cyclodextrins, and the complex, with the process direction being dictated by the value of formation/stability constant (Kf) (Figure 3). The higher the value of this constant, the more stable the inclusion complex [53,54,55].

Cyclodextrins also benefit from commercial availability, biocompatibility, biodegradability, low immunogenicity, lack of toxicity to humans, and ease of functionalization, being excellent nanocarriers for improving the bioavailability of poorly hydrophilic drugs [41,42,50,56]. Additionally, by hosting therapeutic agents, cyclodextrins increase their shelf life, reduce/eliminate their unpleasant taste and odor, and prevent drug–drug or drug–excipient interactions [57,58,59]. Moreover, cyclodextrins’ surfaces can be modified by binding specific ligands that ensure targeted drug delivery to tumor cells in which the correlated receptors are overexpressed while being rarely expressed in healthy cells (e.g., folate, biotin, and glucose receptors) [40]. Another way in which cyclodextrins can achieve targeted delivery of their freight is through controlled degradation of inclusion complexes based on pH changes that result in the loss of hydrogen between the host and guest compounds. Additionally, inclusion complexes may disassemble through heating or enzymatic cleavage of α-1,4 links between glucose units [45].

Thus, cyclodextrin-based delivery vehicles present desirable physicochemical and pharmacokinetic properties without compromising consumer safety [48]. Related to anticancer drugs, these oligosaccharides were reported as efficient carriers for overcoming chemotherapeutics’ poor water solubility and instability, masking their unwanted features, and improving drug bioavailability. Therefore, using cyclodextrin-based oral delivery nanoformulations holds promise for enhancing therapeutic effects against various types of cancer cells while prolonging the lifespan of healthy and regenerative cells [39,48].

Some limitations may hinder the use of cyclodextrins for guest complexation, including the small cavity size of α-cyclodextrin, poor aqueous solubility of β-cyclodextrin, and low productivity of γ-cyclodextrin. Nonetheless, these drawbacks can be overcome through chemical modification (e.g., amination, methylation, etherification, and esterification) of native cyclodextrins, creating derivatives with improved properties [58,60,61]. One more potential issue of cyclodextrin-based medicines is their low complexation efficiency for solid dosage forms, which restricts their application to potent therapeutic agents. Namely, as the drug’s mass increases, the necessary cyclodextrin mass increases, leading to an increased formulation bulk for low-potency drugs. Consequently, a single-dose tablet will not contain a sufficient amount of the drug with low complexation efficiency, whereas larger-diameter tablets can make swallowing more difficult. Therefore, techniques to improve complexation efficiency—such as using water-soluble polymers—are essential for lowering the formulation’s host molecule content and encouraging patient adherence to treatment [62].

## 3. Drug Delivery Systems Based on Chitosan/Cyclodextrins for the Treatment of Gastrointestinal Cancers

Given the appealing characteristics of chitosan and cyclodextrins for designing performant drug delivery vehicles for gastrointestinal cancers, numerous researchers worldwide have investigated these carbohydrate materials, reporting encouraging results. To overview their findings, the following subsections discuss the developments registered with these oligo-/polysaccharides for treating colorectal, liver, gastric, pancreatic, and other digestive system cancers.

### 3.1. Colorectal Cancer

Colorectal cancer (CRC) represents a top killing disease, being the third most frequent form of cancer and the second in terms of death incidence. Thus, it is essential to improve this disease’s prevention, detection, and treatment towards increasing the length and improving the quality of lives of CRC patients [47,63]. Despite chemotherapy being a common treatment choice, this strategy lacks specificity, affecting both tumor cells and rapidly growing healthy cells. Moreover, conventional colon-targeted drug delivery systems are generally degraded and absorbed in the upper gastrointestinal tract before reaching the desired site [28,47,64].

#### 3.1.1. Drug Delivery Systems Based on Cyclodextrins

Numerous recent studies focused on developing colon-specific targeted drug delivery vehicles, with cyclodextrins among the preferred nanocarriers. For instance, Al-Abboodi et al. [65] have prepared an inclusion complex between hydroxypropyl-β-cyclodextrin and clausenidin (Clu/HPβCD) that allowed constant drug release with time and an enhanced drug solubility. Moreover, it was proved that Clu/HPβCD imparted higher cytotoxicity to colon cancer HT-29 cells than the free drug while reducing the effect on normal cells. Concerning the mechanism of action, it was noticed that the inclusion complex triggered reactive oxygen species (ROS)-mediated cytotoxicity in tumor cells, leading to cell cycle arrest and death by apoptosis associated with caspase activation.

Another example is offered by Altoom et al. [66], who have synthesized a β-cyclodextrin/phillipsite composite for the delivery of oxaliplatin. The authors reported significant cytotoxicity in HCT-116 cancer cells, a considerable increase in the cytotoxic effect compared to the free drug, and a controlled release behavior for the chemotherapeutic agent while maintaining a safe profile in normal colorectal cells.

A recent study by Alfassam et al. [67] has investigated the delivery of oxaliplatin and 5-fluorouracil using diatomite’s bio-siliceous frustules functionalized with polymeric chains of β-cyclodextrin as carriers. The results revealed sustained and prolonged drug release (up to 100 h) for the tested delivery system, characteristics further reflected by the enhanced cytotoxic effects on HCT-116 cancer cells.

Differently, Akkin et al. [68] proposed the fabrication of a cyclodextrin nanoplex based on a charge interaction for carrying 5-fluorouracil and Interleukin-2 (IL-2). Drug-loaded nanoplexes exhibited desirable intestinal permeability and higher anticancer activity than free drug solution when tested on CT-26 mouse colon carcinoma cells, demonstrating cumulative release rates of both cargos of more than 80% in 12 h. Thus, the authors concluded that the developed delivery system is a good candidate for cancer treatment advancement, offering a synergistic effect and co-transport of chemotherapeutic drugs and immunotherapeutic molecules while protecting the healthy tissues from unwanted toxicity.

Zhang et al. [69] have developed a supramolecular system [Pt(IV)-SSNPs] based on poly(β-cyclodextrin) for delivering an adamantyl-functionalized platinum(IV) prodrug [Pt(IV)-ADA2]. Their evaluation of the nanocarrier revealed a longer blood circulation time, effective tumor accumulation, successful therapeutic agent uptake by CT-26 cells, cell cycle arrest in G2/M and S phases, apoptosis induction in targeted cells, and insignificant cytotoxicity to major organs.

A different innovative solution is provided by Elamin et al. [70]. The researchers have fabricated a dual targeting supramolecular complex composed of folate-appended methyl-β-cyclodextrin (FA-M-β-CyD) and adamantane-grafted hyaluronic acid. The supramolecular complex exhibited enhanced cytotoxic activity in HCT-1116 cells compared to FA-M-β-CyD alone, benefiting from a more efficient cellular internalization and mitophagy induction in targeted cells. Moreover, when tested in a mouse model of colorectal cancer, the synthesized complex significantly ameliorated the growth of tumor polyps, demonstrating its antiproliferative potential against tumor cells with overexpressed FR-α and CD44 receptors.

**Figure 4:**
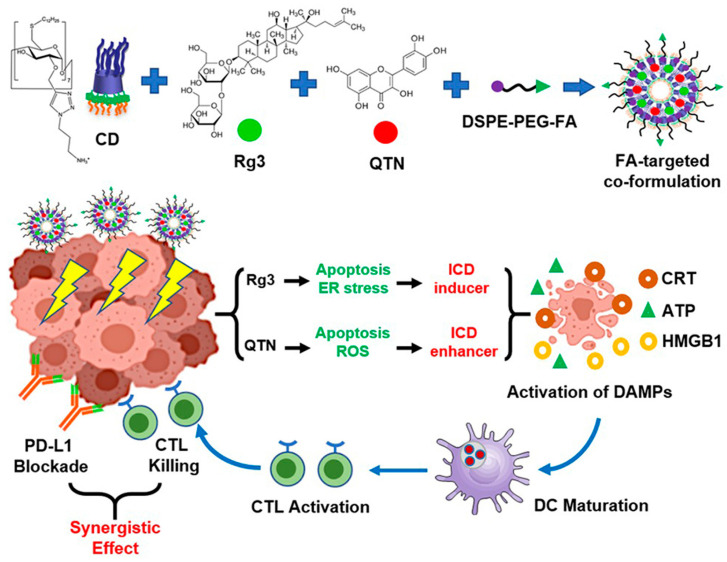
Schematic representation of the delivery system developed by Sun et al. and its mechanism of action. Abbreviations: CD—cyclodextrin; QTN—quercetin; PEG—polyethylene glycol; FA—folate; PD-L1—programmed death-ligand 1; CTL—cytotoxic T lymphocyte; ER—endoplasmic reticulum; ROS—reactive oxygen species; ICD—immunogenic cell death; CRT—calreticulin; ATP—adenosine triphosphate; HMGB1—high-mobility group box 1; DAMP—damage-associated molecular patterns; DC—dendritic cells. Reprinted from an open-access source [71].

Sun et al. [71] have also tackled the advantages of using folate-targeted delivery systems. Specifically, the authors have co-encapsulated ginsenoside Rg3 (Rg3) and quercetin in folate-targeted polyethylene glycol (PEG)-modified amphiphilic cyclodextrin nanoparticles (Figure 4). The obtained nanoformulation considerably extended the blood circulation time and improved tumor targeting in an orthotopic colorectal cancer mouse model. The proposed drug delivery alternative ensured a longer survival of animals in combination with anti-PD-L1, proving its potential for colorectal cancer therapy.

One more folate-targeted delivery strategy is proposed by Zou and colleagues [72], who have created amphiphilic cationic cyclodextrin nanoparticles modified with PEGylated folate loaded with docetaxel and small interfering RNA (siRNA). Thus, a dual freight was carried selectively to colorectal cancer cells, ensuring both chemotherapeutic activity and gene therapy. Explicitly, the nanoformulation enhanced the apoptotic effect of the encapsulated drug by RelA expression downregulation. Hence, the nanosystem could significantly retard tumoral growth without imparting toxicity to normal cells.

Another nanoparticulate formulation is offered by Ünal et al. [73]. The researchers have synthesized cationic nanoparticles for camptothecin encapsulation using two different amphiphilic cyclodextrins coated with polyethyleneimine or chitosan for the nanocarrier preparation. This drug loading strategy allowed for higher cytotoxicity in HT-29 cells compared to the free drug solution, while tests on Caco-2 cells revealed enhanced drug permeability and considerably higher mucosal penetration of the cationic nanoparticle form.

Bai et al. [74] have fabricated channel-type nanoparticles based on host–guest complexes comprising mannose-modified γ-cyclodextrin and regorafenib. In addition to its role as a carrier, the host molecule also played a role in targeting and tumor microenvironment (TME) regulation. The nanoparticles were able to attenuate inflammation and inhibit TAM activation via macrophage targeting and improved the antitumor effect of the included drug via the potentiation of kinase suppression, thus holding promise as a targeted, safe, and effective strategy against colorectal cancer.

Alternatively, Ameli and Alizadeh [75] have employed a pH-responsive acrylic/maleic copolymer combined with β-cyclodextrin for delivering capecitabine to colon cancer cells. The study demonstrated that the prepared delivery vehicles allowed targeted and controlled drug release, liberating the cargo inside the colon in a proportion higher than 80%.

On a different note, Hosseinifar et al. [76] have created a hydrogel-based delivery strategy for 5-fluorouracil by crosslinking alginate with modified β-cyclodextrin. The hydrogels have proven cytocompatible while highly and rapidly accumulating in HT-29 cells and causing a considerable cell death extension by apoptosis compared to free 5-fluorouracil.

An interesting selective drug delivery system has been recently developed by Baek et al. [77]. The researchers proposed the use of a renal-clearable zwitterionic cyclodextrin (i.e., hepatkis-(6-deoxy-6-((phenylboronic acid-tetraethyleneglycol-l-glutamic acid Nα-sulfobetaine)-octaethyleneglycol-caproamide))-β-cyclodextrin) (PBA-(ZW)-CD) for transporting doxorubicin and ulixertinib. The obtained results were promising for colorectal cancer targeting, with the authors reporting enhanced tumor accumulation, facilitated elimination, and improved antitumor efficacy compared to free drugs.

Differently, Fai and colleagues [78] proposed the use of a natural-based drug. Specifically, the authors encapsulated within β-cyclodextrin a hydrogenated active metabolite of curcumin (i.e., tetrahydrocurcumin) and further loaded this inclusion complex in chitosan particles towards create an innovative drug delivery vehicle (THC IC-loaded CPs). When tested against human colon cancer Caco-2 cells, THC IC-loaded CPs displayed an immediate cellular uptake, showing cytotoxicity in a dose-dependent manner.

Low et al. [79] have utilized β-cyclodextrin as host molecules for delivering curcumin to colorectal cancer cells. The authors reported that the inclusion complex significantly decreased cancer cell viability, migration, and invasion rates, while augmenting apoptosis rates in SW480 and HCT-116 cells through caspase 3 activation. It was also observed that the encapsulation strategy improved the aqueous dispersion of curcumin, holding promise for extending its chemotherapeutic application. Additionally, preliminary toxicity results demonstrated the safety of the delivery system in human cancer therapy. Nonetheless, the researchers concluded that further in-depth in vivo studies and clinical trials are required to prove the efficiency against colorectal cancer and other types of malignancies.

One more natural-based alternative is presented by Vukic et al. [80], who have utilized acetylshikonin (AcSh) (i.e., a red pigment from the roots of Boraginaceae family plants) as the guest molecule for β-cyclodextrin host. In comparison to free AcSh, the inclusion complex demonstrated a stronger short-term effect on HCT-116 cells and superior long-term outcomes in both HCT-116 and MDA-MB-231 cell lines, and its effectiveness was correlated with pronounced cell cycle arrest, autophagy inhibition, and enhanced intracellular ROS accumulation.

For clarity, the above-discussed studies have been summarized in Table 1.

#### 3.1.2. Drug Delivery Systems Based on Chitosan

Important recent advancements have also been noted in colorectal cancer-targeted delivery vehicles based on chitosan. For example, Khan and colleagues [81] have developed folate-decorated lipid chitosan hybrid nanoparticles as innovative carriers for 5-fluorouracil that can target HT-29 and HCT 116 cancer cell lines (recognized for their overexpression of folate receptors). The nanosystem allowed for sustained drug release with enhanced chemotherapeutic internalization, resulting in a greater cytotoxic effect on desired cell lines than non-targeted CLPN-2 and the free drug solution. In addition, the safety, biocompatibility, and stability of the presented delivery approach were confirmed by in vivo tests.

**Figure 5:**
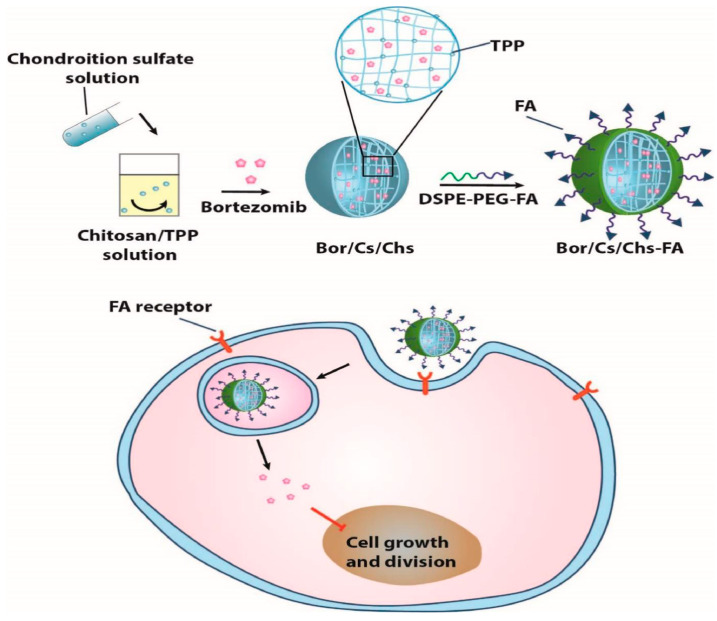
Schematic representation of the fabrication of the nanoparticles designed by Soe et al. and their delivery to folate receptor-expressing colorectal cells. Abbreviations: Bor—bortezomib; Cs—chitosan; Chs—chondroitin sulfate. Reprinted from an open-access source [82].

The same targeting strategy was employed by Soe et al. [82], who fabricated folic acid-conjugated chitosan/chondroitin sulfate self-assembled nanoparticles encapsulated with bortezomib (Figure 5). The researchers reported that this nanosystem permitted selective drug cellular uptake and apoptosis of HCT-116 and HT-29 cells without affecting lung cancer cells (A549), which do not express folate receptors. Thus, the delivery system has been proven effective for chemotherapeutic release to colorectal tumors.

Almeida et al. [83] have designed a delivery system for camptothecin based on micelles made of amphiphilic chitosan modified with PEG and oleic acid. Drug-loaded micelles exhibited significant anticancer effects against HCT-116, Caco-2, and HT-29 colorectal cells in vitro. Moreover, in vivo tests with an HCT-116 xenograft model demonstrated the capacity of the new treatment to considerably reduce tumor growth, while in a more relevant colorectal carcinoma model, it has also been proven to decrease the tumor incidence and inflammation signs.

Another interesting delivery possibility has been presented by Shirani-Bidabadi et al. [84]. The authors have developed chitosan–hyaluronic acid–protamine sulfate polyplexes loaded with a CRISPR/Cas9 plasmid to reverse oxaliplatin resistance in HT-29 cells. The designed system displayed efficient gene delivery, downregulating ERCC1 and restoring drug sensitivity while maintaining negligible toxicity towards healthy cells. Thus, it offers a potential solution for overcoming oxaliplatin resistance.

Alternatively, Sadreddini et al. [32] have created carboxymethyl dextran–chitosan nanoparticles for the co-delivery of doxorubicin and snail siRNA. This novel carrier system demonstrated a significant capacity for downregulating MMP-9 and Vimetin while upregulating E-cadherin in HCT-116 cells. Thus, the treatment with dual-agent nanoparticles produced cell death by apoptosis and migration inhibition in targeted colorectal cancer cells, enhancing its anticancer potential through changes in EMT genes.

Differently, Tian et al. [85] proposed the use of pH-responsive bufadienolide (BU) nanocrystals decorated with a chitosan quaternary ammonium salt. This delivery strategy has proven effective, improving BU internalization, enhancing apoptosis rates, decreasing the mitochondrial membrane potential, and leading to the escalation of ROS levels within tumor cells. Moreover, in vivo experiments revealed effective targeting of intestinal sites, a prolonged retention time, and anti-colon cancer activity via Caspase-3 and Bax/Bcl-2 ratio pathways.

Another pH-responsive system was developed by Narayan and colleagues [86]. Namely, they have constructed mesoporous silica nanoparticles capped with chitosan–glucuronic acid, which they loaded with capecitabine. These colorectal cancer-targeting nanovehicles demonstrated the pH-sensitive and controlled release of the transported drug, achieving higher cellular uptake in HCT-116 cells. Consequently, the nanoparticles could effectively reduce tumors, aberrant crypt foci, dysplasia, and inflammation, while unwanted drug-associated toxicity was diminished.

Hanna et al. [87] have intercalated methotrexate chemotherapeutic in a delivery system composed of poly(3-hydroxybutyrate)/chitosan–graft poly (acrylic acid) conjugated with sodium hyaluronate. The nanocarrier managed to transport the drug in a targeted manner towards Caco-2 cells, ensuring enhanced cytotoxicity through an increase in ROS occurrence and influence on genes related to apoptosis and antioxidant enzymes within treated cells.

Feng et al. [88] have fabricated nanogels made of chitosan and carboxymethyl chitosan to deliver doxorubicin hydrochloride to colorectal cancer cells. Using different crosslinkers for nanogel formation, the authors observed that the materials with a positive zeta potential could be more effectively taken up by tumor cells, significantly reducing their cell viability. Moreover, through their improved mucoadhesion and limited permeability, these nanogels successfully enhanced the contact time of the formulation onto the intestinal mucosa and augmented the local concentration of the therapeutic agent.

A recent study by Bhattacharya et al. [89] focused on developing chitosan–carrageenan nanoparticles for the delivery of imatinib mesylate-poly sarcosine. Based on the experimental results, the authors concluded that these polysaccharide-based nanosystems are promising for the treatment of colon cancer, having great potential in actively targeting and reducing the dose-dependent toxicity of the carried drug.

On a different note, Sorasitthiyanukarn et al. [90] have combined chitosan and alginate into nanoparticles capable of encapsulating curcumin diethyl diglutarate for oral delivery. This natural chemotherapeutic loading approach was reported to have improved physicochemical stability, digestibility, and bioaccessibility under simulated gastrointestinal conditions and cellular uptake in Caco-2 cells.

Another combination of natural polymers for anticancer drug delivery has been reported by Leonard et al. [91]. Specifically, the researchers have synthesized a composite from chitosan and thiolated pectin as a vehicle for 5-fluorouracil. The system was noted to possess superior mucoadhesivity while maintaining selective cytotoxicity (i.e., the delivery system presented targeted cytotoxicity towards HT-29 cells, with milder effects on normal HEK-293 cells).

A more complex delivery vehicle for 5-fluorouracil was proposed by Yusefi and colleagues [92]. The authors have fabricated chitosan-coated magnetic cellulose nanowhiskers. The synthesized nanocomposite displayed desired saturation magnetization and thermal stability, an elevated drug encapsulation capacity, pH-dependent swelling, and fitting drug release performance. Through their appealing physicochemical properties, these nanowhiskers had a high tumor-penetrating ability, presenting a strong activity against colorectal cancer cells.

Another magnetic-based carrier was fabricated by Wu et al. [93]. Explicitly, they created superparamagnetic chitosan-based nanocomplexes able to deliver SN-38 (in the form of the water-soluble polymeric prodrug poly(L-glutamic acid)-SN-38). The developed system could significantly enhance tumor accumulation and ensure cellular internalization through the application of a local magnetic field. This strategy enabled superior targeting and antitumor efficacy, leading to an up to 81% tumor suppression rate in a colorectal cancer model in mice.

Wu et al. [94] have also published a study on colorectal cancer therapeutics, including superparamagnetic nanoparticles. The authors have integrated Fe_3_O_4_ nanoparticles within chitosan-based polyelectrolyte complexes to create a targeted delivery system for irinotecan under a magnetic field. The complexes ensured a high drug encapsulation capacity and demonstrated better anticancer efficacy than the free drug due to improved cell internalization and desirable tumor-targeting ability.

A summary of the mentioned studies concerning chitosan-based delivery vehicles for colorectal cancer treatment is realized in Table 2.

### 3.2. Liver Cancer

Worldwide, liver cancer represents the most common fatal malignancy, posing a major burden on public health [95,96]. Moreover, the poor prognosis of the disease is accentuated by its diagnosis in advanced stages. The conventional treatment route assumes chemotherapy and immunotherapy, yet these approaches exhibit negative effects. Some of their limitations include severe adverse reactions, multiple drug resistance, a high clearance rate, undesired drug distribution to the specific site of liver cancer, and a low concentration of drug that finally reaches liver cancer cells. Therefore, liver cancer patients necessitate better therapeutic alternatives and novel strategies often implying the use of natural compounds and/or nanotechnological approaches [96,97].

#### 3.2.1. Drug Delivery Systems Based on Cyclodextrins

Numerous studies have researched the potential of cyclodextrin-based formulations for chemotherapeutics delivery to liver cancer cells. For instance, Yang et al. [98] have created inclusion complexes between β-cyclodextrin and benzimidazole as promising alternatives for hepatocellular carcinoma. These targeted supramolecular prodrug complexes were able to ensure an accelerated chemotherapeutic release under acidic conditions, allowing efficient uptake into HepG2 cells and subsequently augmented cytotoxicity. Thus, the pH-sensitive system could inhibit liver cell proliferation by inducing cell apoptosis.

Wei et al. [99] have also fabricated a delivery system with a drug-liberating capacity in acidic environments. The researchers have utilized a pH-responsive cyclodextrin derivative (R6H4-CMβCD) for creating nanoparticles suitable for curcumin encapsulation and targeted transport. These nanocarriers were noted to improve cellular uptake and ensure enhanced accumulation in hepatoma cells. Additionally, the nanoparticles could avoid lysosome action via the “proton sponge effect”, producing higher apoptosis rates and excellent antitumor outcomes without affecting other major organs.

A different targeting method was proposed by Wu and colleagues [100]. Specifically, the authors have developed light/redox dual stimuli-responsive β-cyclodextrin-gated mesoporous nanoparticles functionalized with an azobenzene/galactose-grafted polymer as an innovative doxorubicin carrier to hepatocellular carcinoma cells. This nanosystem allowed the controlled and targeted release of its freight, ensuring a more efficient delivery into HepG2 cells and enhanced cytotoxicity compared to HeLa and COS7 cells.

A study conducted by Fan et al. [101] has focused on the encapsulation of doxorubicin into folic acid–polyethylene glycol–β-cyclodextrin nanoparticles. The developed delivery system improved drug solubility ensured controlled medicine release, and allowed targeted drug delivery to HepG2 cells while maintaining desirable blood compatibility. Thus, it offers great promise for improving liver cancer treatment.

A similar strategy was tackled by Li and colleagues [102], who have loaded melarsoprol into folate-targeted polyethylene glycol-modified amphiphilic cyclodextrin nanoparticles. This nanoformulation ensured cell-specific uptake, cytotoxicity, apoptosis, and migration inhibition in hepatocellular carcinoma cells, effects that further contributed to prolonging tested animals’ survival without imparting toxicity to healthy organs.

Alternatively, Bognanni et al. [103] have designed cross-linked γ- and β-cyclodextrin polymers as carriers for doxorubicin and oxaliplatin. Moreover, γ-cyclodextrin molecules have been functionalized with arginine–glycine–aspartic acid or arginine moieties to target integrin receptors from tumoral cells. When tested against liver and lung carcinoma cell lines it was found that the developed system could considerably enhance the antiproliferative activity of doxorubicin in HepG2 cells only, while the cytotoxicity of oxaliplatin was increased in both cell lines. The improved anticancer effects were correlated with a higher accumulation of the chemotherapeutic inside the cells, while the functionalization strategy resulted in no additional effect compared to the precursor polymer.

Yang et al.’s research group [104,105] focused on grafting pullulan to β-cyclodextrin to create an efficient doxorubicin nanocarrier for liver-specific delivery. For example, they have synthesized glycyrrhetinic acid–β-cyclodextrin grafted pullulan nanoparticles that allowed slow drug release, high cellular uptake, and better therapeutic outcomes [104]. Another drug delivery system developed by the same research group [105] consists of biotinylated β-cyclodextrin-grafted pullulan. These doxorubicin-loaded nanoparticles could inhibit tumor cell growth, given their enhanced accumulation in the liver, while the cardio–renal toxicity was considerably reduced.

Another interesting doxorubicin carrier was proposed by Daga and colleagues [106], who have loaded this drug into GSH-responsive cyclodextrin-based nanosponges. These delivery systems displayed a good safety profile, with comparable cytotoxicity and hepatic accumulation to free doxorubicin. Moreover, the developed nanosponges were successfully taken up through active mechanisms and were able to escape the efflux drug pump, thus aiding in circumventing drug resistance.

One more innovative nanovehicle of interest for liver cancer treatment has been constructed by Wen et al. [107]. The authors have coated a β-cyclodextrin-cholic acid–hyaluronic acid polymer onto magnetite-graphene oxide and further loaded the nanomaterial with camptotechin. These multiple targeted features allowed for a strong antitumor effect, given that the chemotherapeutic action worked in synergy with the photothermal activity of the nanomaterial towards inhibiting liver cancer cell growth. Specifically, in addition to their drug-release capacity, the nanocomposites induced local hyperthermia that produced tumor cell apoptosis under near-infrared radiation.

On a different note, Ercan and colleagues [108] have elaborated blank self-assembled polycationic amphiphilic β-cyclodextrin nanoparticles and tested their activity against HepG2 cells. Without encapsulating any drug, the developed nanoformulation could exert anti-proliferative activity on hepatocellular carcinoma cells, triggering apoptosis and restoring tumor cell chemosensitivity.

The above-discussed studies have been synthesized in Table 3 to offer a clearer perspective on the recent developments in cyclodextrin-based drug delivery systems for liver cancer.

#### 3.2.2. Drug Delivery Systems Based on Chitosan

Several recent promising anti-liver cancer formulations have also been reported to be based on chitosan. As an example, Ye et al. [109] have encapsulated doxorubicin into chitosan- and folic acid-functionalized chitosan nanoparticles. The two nanomaterials exhibited similar drug release rates. However, the use of the targeting agent resulted in higher cytotoxicity levels, promoting apoptosis, arresting the cell cycle at the G2/M phase, and upregulating p53.

Song and colleagues [110] have recently synthesized multifunctional thiolated chitosan derivatives, which they further loaded with arsenic trioxide through glutathione-sensitive bonds. This structure allowed the drug to be released in a proportion of 95% after 24 h in the glutathione environment, while only low leakage was noted in physiological conditions. Therefore, the nanocarrier permits a targeted release, resulting in enhanced tumor intracellular accumulation of the transported chemotherapeutics while reducing unwanted adverse effects on healthy organs. Moreover, when tested on the HepG2 mouse tumor model, the nanosystem proved highly effective in treating liver cancer, inducing an 86.4% tumor inhibition rate.

An alternative targeting vehicle was proposed by Yan et al. [111]. The authors have fabricated a redox-responsive micelle for doxorubicin and pheophorbide A based on poly-ε-caprolactone linked to carboxymethyl chitosan through a disulfide bond and functionalized with the glycyrrhetinic acid targeting ligand. This complex system could effectively extend the average residence time in circulation, leading to enhanced intracellular uptake by HepG2 cells. Moreover, the developed nanoplatform could exert synergistic activity with the carried drugs, improving inhibition efficiency and endowing the system with photo–chemo theranostic and NIR imaging capabilities.

Differently, several research groups have focused their studies on the delivery of natural-based chemotherapeutic agents. For instance, Huang et al. [112] have loaded curcumin into galactosylated chitosan-modified nanoparticles based on PEG-PLGA for targeting asialoglycoprotein receptor (ASGPR) expressed on hepatocellular carcinoma cells. The fabricated nanosystems were effectively internalized by HepG2 cells, successfully accumulating and releasing curcumin within tumor sites. Thus, the nanocarriers offered a superior tumor growth inhibitory potential compared to the free drug while maintaining excellent biocompatibility with normal tissues.

Sorasitthiyanukarn et al. [113] have alternatively employed chitosan/alginate nanoparticles for the delivery of curcumin diglutaric acid, given its better solubility and antinociceptive effects compared to curcumin. The developed nanoparticles exhibited slow cumulative release of the incorporated agent, and the release pattern was attributed to Fickian diffusion and the erosion of carrier polymeric materials. The nanosystem demonstrated higher in vitro cellular uptake in Caco-2 cells and better antitumoral activity against Caco-2, HepG2, and MDA-MB-231 cancer cells, holding promise as a useful tool in future oral-administered anticancer therapeutics.

One more natural-based possibility has been envisaged by Yang and colleagues [114], who have created a delivery system for zedoary turmeric oil (ZTO). Namely, the authors have encapsulated ZTO into chitosan-coated solid lipid nanoparticles, obtaining a promising delivery platform for this otherwise volatile and insoluble agent. The researchers reported that the use of the chitosan coating resulted in higher liver accumulation compared to uncoated particles, leading to significantly improved bioavailability and enhanced cellular internalization.

A different natural chemotherapeutic was used by Zhang et al. [115]. Specifically, the researchers have loaded ginsenoside compound K into micelles made of deoxycholic acid–O-carboxymethyl chitosan and A54 peptide. This drug transport system ensured a pH-responsive and sustained release behavior, allowing a significantly stronger in vitro cytotoxicity against HepG2 and Huh-7 cells than the free chemotherapeutic. Moreover, the developed micelles could promote the protein expression levels of caspase-3, caspase-9, and poly (ADP-ribose) polymerase, augmenting anticancer activity.

An innovative chitosan-based formulation has also been proposed by Harisa et al. [116]. The research group has synthesized erythrocytes loaded with pravastatin–chitosan nanogels that were able to maintain a sustained drug release over 48 h. The nanosystem could reduce the cellular viability of HepG2 cells by 28% compared to unloaded erythrocytes, showing good promise for the targeted treatment of liver cancer.

For clarity, Table 4 presents the above-detailed studies in a more concise manner.

### 3.3. Gastric Cancer

Gastric cancer represents the 4th leading origin of tumors and the 3rd most frequent cause of cancer-related deaths [117]. Other unfortunate characteristics of gastric cancer comprise high incidence rates of metastasis and low rates of early diagnosis, radical resection, and 5-year survival. The usual treatment route assumes radical surgery followed by chemotherapy in patients diagnosed with gastric cancer in early disease stages, a therapeutic strategy that generally results in a 90% survival rate in 5 years after the intervention. However, patients with advanced gastric cancer stages do not have the possibility of surgery and have a high metastasis risk; these factors lead to a poor prognosis. Therefore, developing performant unconventional drug delivery systems is in high demand for creating an efficient treatment for gastric cancer [12,118].

Several studies have been conducted on the encapsulation into oligo-/polysaccharides of certain drugs of interest for gastric cancer. For example, Gaur et al. [119] have reported on the preclinical efficacy of CRLX101, a nanoparticulate structure containing a cyclodextrin-based polymer and camptothecin. In vitro tests demonstrated high cytotoxicity against gastric cancer cell lines, while in vivo studies registered potent antitumor activity. In addition, there was a significant decrease in the expression of the carbonic anhydrase, VEGF, and CD31 proteins in treated tumors, proving hypoxia and angiogenesis inhibition.

On a different note, other researchers have directed their efforts to create innovative drug delivery vehicles based on chitosan. One such case is represented by the study by Wu and colleagues [117], who have designed and fabricated cholesterol-loaded chitosan nanoparticles for the delivery of salinomycin and siRNA (siRNA@C-SAL). The siRNA@C-SAL was able to induce superior cytotoxicity in SNU-668 and SGC-791 cells without causing any significant adverse effects on healthy organs. Moreover, no weight loss was observed when using this treatment in tumor-bearing mice, reconfirming the safe profile of this formulation.

An alternative strategy was recently proposed by Bandi et al. [120]. The researchers have developed a multi-layered mucoadhesive gastric patch (based on a chitosan–hydrocaffeic acid conjugate) for the delivery of regorafenib. When tested in a rat model, the fabricated patches ensured a constant plasma drug concentration, sustaining its release for 8 days after oral administration. This delivery approach resulted in a significant tumor volume reduction in athymic nude mice over 7 days, recommending the platform as a long-acting oral drug delivery system.

Differently, Jiang et al. [121] have prepared chitosan oligossacharide-conjugated selenium (COS-Se) as a novel anticancer therapy. The unconventional chemotherapeutic could enhance phagocytosis and the secretion of anti-inflammatory cytokines in mouse peritoneal macrophages. Furthermore, COS-Se produced a considerable immuno-enhancing effect by promoting the phagocytic, spleen, and thymus indexes without imparting cytotoxicity to normal cells. It also significantly inhibited the proliferation and migration of gastric cancer cells, remarkably repressing gastric adenocarcinoma growth while keeping a nontoxic activity towards normal fibroblast cells.

An interesting material proposal was also made by Zhang et al. [122], who utilized N-deoxycholic acid glycol chitosan as the carrier and a gastric cancer angiogenesis marker peptide (i.e., GX1) conjugated with PEG–deoxycholic acid as the targeting ligand for fabricating a drug vehicle suitable for docetaxel delivery. This innovative nanostructure allowed sustained drug release accelerated by an acidic pH, a liberating behavior that enhanced cellular uptake and resulted in stronger cytotoxicity against co-cultured gastric cancer cells and human umbilical vein endothelial cells than the free drug. In addition, the delivery system significantly inhibited tumor growth in SGC791 tumor-bearing mice without producing any weight loss in treated animals.

Alternatively, Chi et al. [123] have prepared novel polymer–drug conjugates from carboxymethyl chitosan and norcantharidin. The developed vehicles could significantly reduce the systemic toxicity of the carried drug while enhancing its antitumor efficacy in vivo. Explicitly, this innovative delivery system produced a 59.57% tumor suppression rate against SGC-7901 gastric tumors in BALB/c nude mice, an anticancer activity that was further correlated with the upregulation of TNF-α and Bax and downregulation of VEGF, Bcl-2, MMP-2, and MMP-9 expression. Thus, these conjugates are promising and feasible therapeutic options for managing gastrointestinal tumors by inhibiting tumor metastasis and inducing apoptosis in vivo.

**Figure 6:**
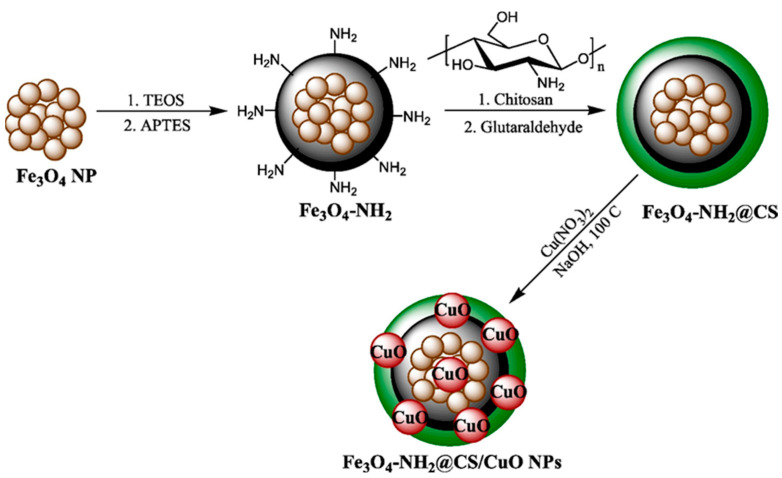
Preparation of the Fe_3_O_4_-NH_2_@CS/CuO magnetic nanocomposite. Reprinted from an open-access source [124].

Wang et al. [124] have rather focused on the anticancer properties of metal oxide nanoparticles. Explicitly, the authors have obtained chitosan-modified amino-magnetic nanoparticles for supporting copper oxide nanoparticles (Figure 6). The as-described nanocomposite leads to the very low cell viability of human gastric and colorectal carcinoma cell lines, showing particular promise for treating gastro-duodenal cancers. The high anticancer effect was correlated with the desirable antioxidant activity of the system.

Moving to natural chemotherapeutic agents, Issarachot et al. [125] have recently created superporous hydrogels made of chitosan–PVA blends for the delivery of a resveratrol solid dispersion. The authors reported an efficient drug release sustained over 12 h. The formulation showed slightly less cytotoxicity towards AGS cells than pure resveratrol while displaying a similar anti-inflammatory activity against RAW 264.7 cells to indomethacin.

Another interesting example is offered by Catchpole et al. [126], who created inclusion complexes between New Zealand propolis and α-, ß-, and γ-cyclodextrins. The formulated complexes displayed strong proliferation-inhibitory activity against four human gastrointestinal cancer cell lines, including gastric carcinoma cells. The delivery systems had also reportedly exerted potent anti-inflammatory and lipid antioxidant activities, effects that were not only correlated with the encapsulated propolis components (i.e., flavonoids, phenolic acids, and caffeate-type esters) but also with the synergism between the carrier cyclodextrins and these bioactive agents.

The above-discussed studies have been summarized in Table 5 to provide an at-glance perspective on the novelties of cyclodextrin and chitosan-based drug delivery systems for gastric cancer.

### 3.4. Pancreatic Cancer

Pancreatic cancer is one of the most lethal cancers, as it has an aggressive metastatic progression with less than a 10% 5-year survival rate after diagnosis. Being asymptomatic in the early stages, this disease is often discovered quite late. Moreover, pancreatic cancer treatment is impeded by the inaccessible anatomical position of this organ and the inability of chemotherapeutic agents to penetrate the dense extracellular matrix surrounding pancreatic tumor cells [9,127,128,129]. This unfortunate context has led to the prediction that, by 2030, pancreatic cancer will become the second leading cause of cancer death in the absence of better-suited therapeutics [9].

#### 3.4.1. Drug Delivery Systems Based on Cyclodextrins

Thus, researchers have concerted their efforts towards developing efficient delivery systems that would allow the carried drugs to reach target tumor sites [128]. Tackling the recognized benefits of cyclodextrins, scientists have elaborated several formulations based on these oligosaccharides that showed encouraging results.

Kano et al. [130] have recently synthesized cyclodextrin-conjugated α-bisabolol and tested it against pancreatic cancer cells. The authors reported significant modifications of cytomorphology, apoptosis induction, and the suppression of phosphorylation of focal adhesion kinase. Moreover, testing this treatment in subcutaneous xenograft models reduced the tumor volume compared with control groups and lower Ki67-positive cells than in gemcitabine-treated groups. The research concluded that this formulation could improve the prognosis of pancreatic cancer patients, yet further investigations should be performed to determine the precise mechanisms of its antitumor effects to facilitate subsequent clinical applications.

Alternatively, Iacobazzi et al. [131] have created stable complexes between hydroxy-propyl-β-cyclodextrin as host molecules and PTA34 and PTA73 as guests. The results obtained through in vitro studies revealed that the formed complexes had high antitumor activity against pancreatic ductal adenocarcinoma (PDAC) cells, leading to a strong G2/M phase arrest followed by the induction of apoptosis. More recently, Bhattacharyya et al. [132] have used the same host compounds for creating improved PDAC therapeutics. Nonetheless, the researchers used difluorinated curcumin as the guest molecule, obtaining complexes with increased antiproliferative activity. The anticancer effects of the inclusion complex were reflected in its ability to inhibit colony and spheroid formation and capacity to induce cell cycle arrest and apoptosis in PDAC cells.

On a different note, Higashi et al. [128] have mixed adamantane-modified bromelain and multisubstituted-PEGylated β-cyclodextrins to create a drug delivery system for pancreatic cancer. The resulting host–guest complexes exhibited long blood retention and high tumor accumulation, providing strong antitumor activity. Moreover, this drug formulation also worked as an enhancer of the anticancer effects of conventional chemotherapeutics if pre-administered.

An interesting treatment alternative has been proposed by Dora et al. [133]. Namely, the authors have prepared β-cyclodextrin nanosponges loaded with erlotinib and tested their potential against pancreatic cell lines (i.e., MIA PaCa-2 and PANC-1). A higher intracellular uptake was noted for the nanoformulation compared with the free drug, leading to improved toxicity in the target cells. Furthermore, the increased solubility, dissolution, and oral bioavailability of the chemotherapeutic agent in this form allow drug dose reduction and the subsequent limitation of dose-related adverse effects.

The discussed studies have been summarized in Table 6.

#### 3.4.2. Drug Delivery Systems Based on Chitosan

Several other studies have focused on enhancing the activity of anticancer drugs by integrating them into chitosan-based nanoformulations [127]. For instance, David and colleagues [134] have used chitosan nanoparticles for the co-delivery of quercetin and 5-fluorouracil. The authors reported encouraging results, with the dual-loaded delivery system displaying considerable toxicity towards the primary pancreatic cancer cell line, MIA PaCa2, in both 2D and 3D cultures.

Differently, Zhou et al. [135] have fabricated folate–chitosan–gemcitabine core-shell nanoparticles and evaluated their effects against metastatic pancreatic adenocarcinoma cells (i.e., COLO357). The authors reported that the developed nanosystem significantly inhibited target cell proliferation and accumulated in human pancreatic cancer xenografts, while the delivery systems without functionalization agents were mainly found in normal liver tissues.

Zeng et al. [136] have proposed the utilization of a celastrol–chitosan oligosaccharide conjugate for pancreatic cancer drug delivery. The nanocarrier system considerably inhibited tumor growth, induced apoptosis, and suppressed tumor metastasis while keeping reduced cytotoxicity towards hepatic cells than free celastrol. Considering that the developed formulation could increase antitumor efficacy, prolong the drug circulation time, and reduce subacute toxicity, the authors concluded that it holds promise as an alternative treatment for pancreatic cancer patients.

Another chitosan-based delivery system has been recently developed by Naeeni et al. [137]. Specifically, the researchers have encapsulated a natural bioactive compound (i.e., lawsone) into a liposomal nanoparticle coated with chitosan–folate. The nanosystem presented strong free radical scavenging activity and could significantly inhibit pancreatic cancer cell proliferation. In addition, it notably enhanced cellular uptake and considerably upregulated the Caspase 3, 9, and Bax genes responsible for apoptosis.

Thakkar and colleagues [138] have alternatively fabricated chitosan-coated solid lipid nanoparticles loaded with ferulic acid and aspirin as a pancreatic cancer chemopreventive strategy. The dual cargo delivery system significantly reduced cell viability in MIA PaCa-2 and Panc-1 cell lines, increasing apoptosis rates in the target cells and suppressing tumor growth. Moreover, an immunohistochemical analysis of tumor tissues revealed lowered expression of the proliferation proteins PCNA and MKI67, while the apoptotic proteins p-RB, p21, and p-ERK1/2 had increased expression levels.

For clarity, Table 7 summarizes the above-discussed studies, offering a concise view of promising chitosan-based drug delivery systems for pancreatic cancer.

### 3.5. Other Cancers of the Digestive System

In addition to the above-discussed cancer types, several advancements have been reported for other malignant diseases of the digestive system. The few identified studies reporting on the use of chitosan- and cyclodextrin-based delivery systems for digestive cancers outside the gastrointestinal tract are further described in this subsection.

**Figure 7:**
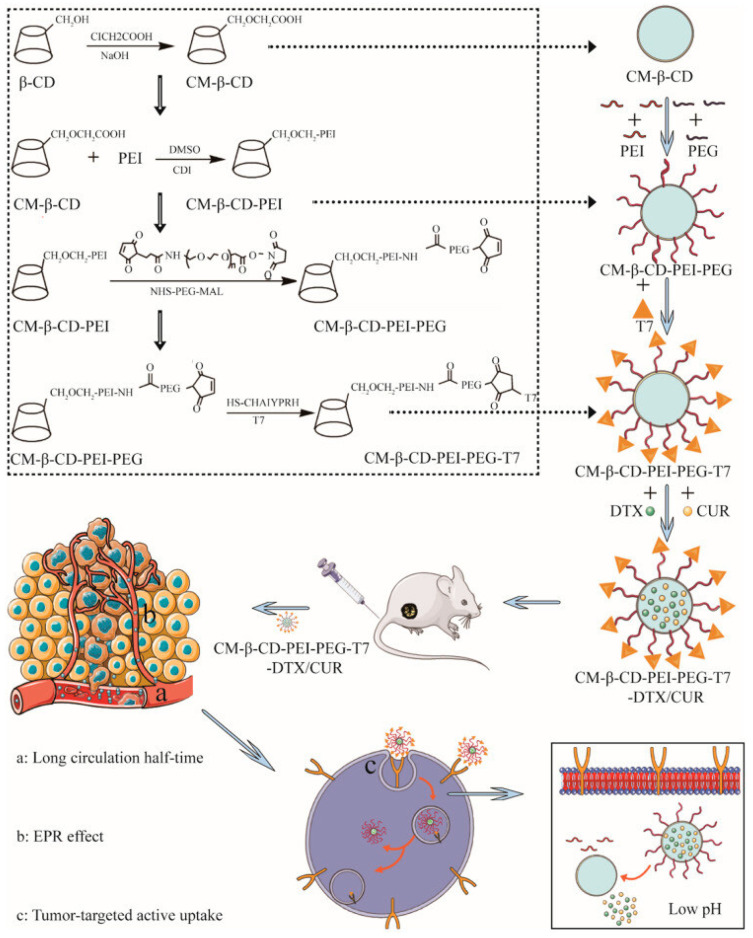
Schematic representation of the preparation of the CM-β-CD-PEI-PEG-T7 copolymer utilized by Deng et al. Reprinted from an open-access source [139].

A study by Deng et al. [139] has reported on fabricating a novel T7 peptide-modified pH-responsive targeted nanosystem co-loaded with curcumin and docetaxel for treating esophageal cancer (Figure 7). This complex nanocarrier could effectively transport a dual freight, ensuring drug release in a pH-responsive manner, enhancing cellular uptake, and improving growth suppression in a KYSE150 esophageal cancer model. Moreover, synergistic antitumor activity was observed, confirming once more the potential of this combined therapy for esophageal cancer.

A recent drug delivery system against esophageal cancer was also proposed by Su and colleagues [140]. The researchers have synthesized inclusion complexes between curcumol and β-cyclodextrin and tested their efficacy in parallel with radiation administration in vitro and in vivo. The in vitro evaluation revealed synergistic anticancer effects, noting inhibited proliferation, reduced colony formation, increased apoptosis, inhibition of DNA damage repair, and radiosensitization of esophageal cancer cells. In vivo tests further confirmed a stronger antitumoral activity of the combined therapeutic approach than for each monotherapy alone.

Additionally, one of the mentioned cyclodextrin-based formulations [119] that was previously preclinically tested against gastric cancer has also reached the stage of human testing. Specifically, clinical trial NCT01612546 [141] has employed the CRLX101 nanopharmaceutical for treating patients with advanced or metastatic stomach, gastroesophageal, or esophageal cancer. The enrolled people had unresectable tumors that had been previously treated with at least one regimen of chemotherapy. CRLX101 was used to deliver the cytotoxic topoisomerase-1 inhibitor camptothecin into tumor cells to interrupt their growth. This study’s approach assumed nanoformulation administration for more than 60 min on days 1 and 15 at 15 mg/m^2^, a treatment that was repeated every 28 days for six courses in the absence of disease progression or unacceptable toxicity. The patients who reached a stable phase of the disease or were in better condition after completing the six courses could further receive 6 months of additional treatment.

Several studies worth mentioning have focused on chitosan-based formulations. For instance, Hu et al. [142] have elaborated nanocomposites made of chitosan–sodium alginate–polyethylene glycol–crocin and investigated their effects on esophageal cancer KYSE-150 cells. The proposed unconventional therapeutic decreased the viability of tumor cells without affecting normal Het-1A cells. Furthermore, the nanosystem augmented ROS production, decreased MMP levels, induced apoptotic cell death, inhibited the migration of KYSE-150 cells, and decreased GSH and SOD activity.

Differently, Mazzarino et al. [143] have concentrated their efforts on improving oral cancer treatment. In this respect, the researchers have created an innovative delivery system using polycaprolactone nanoparticles coated with chitosan encapsulating curcumin. This formulation has reportedly produced a considerable reduction in SCC-9 human oral cancer cell viability in a concentration- and time-dependent manner.

Graciano et al. [144] have alternatively created chitosan gels loaded with toluidine blue O, a photosensitizer with potential in photodynamic therapy. The carrier system had desirable features for buccal delivery, enhancing photosensitizer retention in the oral mucosa and inducing apoptosis after laser irradiation.

One more interesting therapeutic alternative for oral cancer treatment was proposed by Mariadoss and colleagues [145], who have encapsulated phloretin into chitosan nanoparticles. These nanosystems were registered to enhance the mitochondria-mediated apoptotic mechanism as they stimulated ROS production, depletion of cellular antioxidants, and cell cycle arrest.

### 3.6. Summative Discussion

The recent efforts of scientists worldwide have materialized in a series of oligo-/polysaccharide-based drug delivery systems with great promise in treating various digestive system cancers. Being able to transport a wide range of chemotherapeutics, chitosan and cyclodextrin-based carriers have been exploited in numerous studies aimed at finding improved anticancer strategies, especially against colorectal, liver, gastric, and pancreatic malignant tumors.

Chitosan and cyclodextrins exhibit unique intrinsic properties of interest for designing gastrointestinal-specific drug delivery systems. Specifically, chitosan benefits from preferential protonation and solubilization in acidic environments, has a high zeta potential that enables effective interactions with the negatively charged membranes of cancer cells and endothelial cells of the tumor vasculature, and can transiently open the tight junctions between epithelial cells, increasing the cellular uptake probability of delivered chemotherapeutics [16,20]. On the other hand, cyclodextrin-based inclusion complexes allow controlled degradation with pH changes (losing the hydrogen bonds between host and guest molecules) or in the presence of heat/enzymes that lead to α-1,4 link cleavages between glucose units [45]. In addition, utilizing cyclodextrins in drug formulations has the advantage of enabling selective tumor uptake due to neoplastic cells’ high glucose consumption [146].

Moreover, the ease of functionalization of these carbohydrate materials allowed researchers to create targeted formulations that can release drugs in response to certain stimuli. Among the preferred modifications is the creation of folic acid-conjugated systems that can target tumoral cells with overexpressed folate receptors, a strategy that was tackled in many studies focused on both cyclodextrin- [70,71,72,101,102] and chitosan-based carriers [81,82,109,135,137]. A frequent stimuli-responsive option is also the generation of pH-sensitive formulations, which have been found to be used against most cancer types. Numerous research teams have successfully fabricated pH-responsive cyclodextrin [59,82,83] and chitosan-based drug delivery systems [69,70,99,105], leading to promising in vitro and in vivo results. Another common approach consists of developing hyaluronic acid-grafted systems that reach desired sites by targeting CD44 receptors on tumor cells. This targeting strategy has been considered for several cyclodextrin- [70,107] and chitosan-based delivery vehicles [84]. The enhanced mucoadhesion of chitosan-based systems was also considered an efficient alternative for augmenting the local concentration of encapsulated drugs for colorectal cancer [88,91] and gastric cancer [120]. Chitosan has also been reported as a component of magnetic composite materials, permitting the development of delivery systems that can be guided by an external magnetic field for reaching colorectal [92,93,94] and gastric tumors [124]. Several targeting alternatives among the reported studies were noted to be specific for liver cancer, including redox-responsive delivery systems [100,111], GSH-responsive formulations [106,110], carriers grafted with a glycyrrhetinic acid targeting ligand [104,111], vehicles functionalized with arginine–glycine–aspartic acid or arginine moieties to target integrin receptors [103], and galactosylated carriers for targeting asialoglycoprotein receptor (ASGPR) expressed on hepatocellular carcinoma cells [112]. In contrast, a distinct targeting option for gastric cancer assumed the utilization of angiogenesis marker peptide (i.e., GX1) conjugated with PEG–deoxycholic acid as the targeting ligand [122].

Concerning the administration routes for the developed formulations, the most common approaches were oral and intravenous delivery possibilities, with the preference between the two depending on the carrier material and cancer type. Specifically, in the case of colorectal cancer, cyclodextrin-based drug delivery vehicles were generally designed and tested for intravenous administration, being usually grafted with the aforementioned ligands for actively targeting overexpressed receptors on tumor cells [69,70,71,72,77]. Several formulations have also been developed for oral administration [65,68,73,75]; however, so far, they have been only tested in vitro, and it can only be assumed that they will be able to protect the therapeutic cargo throughout the gastrointestinal tract before reaching tumors in the colon/rectum, requiring in vivo studies to confirm these hypotheses. The opposite situation was observed for chitosan-based vehicles employed in colorectal cancer treatment, as the mucoadhesion and pH-sensitivity of this material attracted more interest towards developing orally administered chemotherapeutics [83,85,86,90,92]. On the other hand, creating chitosan-based delivery vehicles for intravenous administration imposed different targeting strategies, such as the use of folic acid to ensure selectivity towards cells with overexpressed folate receptors [82], loading with an imatinib mesylate drug that acts as a tyrosine kinase inhibitor, with specific targets of BCR-ABL and c-KIT kinases [89], and creating composites with magnetic nanoparticles to be guided under the application of an external magnetic field [93,94]. Regarding gastric cancer, only one of the proposed formulations was designed for oral administration considering the mucoadhesion of chitosan [120], whereas other carriers were developed to be injected intravenously (with targeting based on chitosan’s intrinsic properties [117,123] and the addition of GX1 as a targeting ligand [122]), intraperitoneally [119], and subcutaneously [121]. In what concerns liver and pancreatic cancers, their sites are not located in the digestive tube. Thus, the preferred administration route was observed to be intravenous injection. Specifically, in the case of cyclodextrin-based delivery vehicles for liver cancer, most formulations assumed intravenously administered carriers either functionalized with active targeting ligands (i.e., folic acid [101,102], biotin [105], glycyrrhetinic acid [104], and hyaluronic acid [107]) or GSH-responsive systems [106]. Only one formulation was developed for oral administration characterized by pH-responsive drug delivery and increased gastrointestinal stability [99]. On the other hand, chitosan-based systems for intravenous injection against liver cancers were endowed with GSH-responsive [110], redox-responsive [111], and active ASGPR-targeting properties [112]. One study also reports using an oral chitosan-based formulation that ensured desirable stability in a simulated gastrointestinal environment, with slow cumulative drug release displayed in simulated gastrointestinal fluids without enzymes and in body fluid [113]. As for pancreatic cancer, cyclodextrin-based formulations were reported for intravenous injection [128,130], oral delivery [133] (demonstrated by in vivo studies to facilitate absorption and avoid pre-systemic metabolism, increasing the bioavailability of the carried drug), and potential suitability for both oral and parenteral administration [131] (only tested in vitro). Differently, among the chitosan-based formulations designed for a specific type of administration for fighting pancreatic cancer, one was engineered for intravenous delivery via folate receptor targeting [135], and two were developed for oral administration based on the intrinsic properties of this natural polymer [136,138]. Both of these were able to prolong the circulation time of loaded drugs and ensure desirable intestinal absorption.

Despite the encouraging results obtained through in vitro and in vivo tests, there is a long way until the described formulations can enter the market. Until now, there has only been one study that reached clinical testing, while the other developed formulations require more in-depth investigations before being tested on humans. Moreover, from the point of view of their fabrication, the discussed delivery systems have only been produced at a laboratory scale. Thus, moving towards large-scale production assumes a critical step for technology transfer, yet it requires a long list of considerations. Scaling up to industrial manufacturing is challenging, especially regarding the reproducibility, controlled production, targetability, and functionality of delivery nanosystems, green synthesis routes, contamination risks, complex stepwise operations, safety concerns, and cost-effectiveness [147,148,149].

## 4. Conclusions and Future Perspectives

To summarize, innovative drug delivery vehicles with high anticancer performance can be obtained by taking advantage of the unique physicochemical properties of cyclodextrins and chitosan. Through their efficiency in carrying chemotherapeutic agents to the targeted areas of the digestive system, these oligo- and polysaccharides hold much promise in developing better treatment solutions for colorectal, liver, gastric, pancreatic, esophageal, and oral cancers. The versatility of these carbohydrate materials in terms of functionalization and encapsulation possibilities offers an alternative to conventional chemotherapeutic administration with great potential.

Numerous chitosan- and cyclodextrin-based formulations have been tested in vitro and in vivo, leading to encouraging results, such as prolonged circulation times, improved cellular internalization of carried drugs, preferential uptake by the targeted cells, reduced side effects, enhanced apoptosis rates, and increased tumor suppression rates. Moreover, some nanocarriers were reported to work in synergy with the transported natural or synthetic chemotherapeutic, augmenting its anticancer activity or sensitizing otherwise drug-resistant cells.

Nonetheless, the vast majority of reviewed studies have only achieved preclinical testing stages. There is only one clinical trial on a cyclodextrin-based formulation for stomach, gastroesophageal, and esophageal cancer treatment. All the other proposed delivery systems have proven effective against gastrointestinal cell cultures or in small laboratory animals affected by this group of diseases. Thus, given the importance of finding better-performing anticancer therapeutics, the multitude of novel drug delivery systems should be researched in more depth, in animals more similar to humans, and then in clinical trials to ensure their rapid translation to clinical settings.

To conclude, by overviewing the most recent studies in the field and creating an updated background of what concerns chitosan-/cyclodextrin-based drug delivery systems for gastrointestinal cancers, this paper hopes to serve as an inception point for further research and technological advancements to widen cancer treatment possibilities.

## Figures and Tables

**Table 1:** Overview of cyclodextrin-based drug delivery systems for colorectal cancer.

Drug Delivery System	Carried Agent(s)	Main Observations	Ref.
Hydroxypropyl-β-cyclodextrin	Clausenidin	Greater cytotoxic effect on colon cancer HT-29 cells than the free drug Treated HT-29 cells displayed cell cycle arrest and death by apoptosis Reduced side effects	[65]
β-Cyclodextrin/phillipsite composite	Oxaliplatin	Greater cytotoxic effect on colon cancer HCT-116 cells than the free drug Controlled release behavior Safe in normal colorectal cells	[66]
Diatomite’s bio-siliceous frustules functionalized with polymeric chains of β-cyclodextrin	Oxaliplatin and 5-fluorouracil	Greater cytotoxic effect on colon cancer HCT-116 cells than free drugs Significant sustained and prolonged drug release	[67]
Cyclodextrin nanoplex	5-fluorouracil and IL-2	Greater cytotoxic effect on colon cancer CT-26 cells than the free drug solution Suitable intestinal permeability for oral administration	[68]
Supramolecular complex composed of [Pt(IV)-SSNPs] based on poly(β-cyclodextrin)	Adamantyl-functionalized platinum(IV) prodrug [Pt(IV)-ADA2]	Effective tumor accumulation and negligible cytotoxicity to major organs Greater cytotoxic effect on colon cancer CT-26 cells than the free drug solution Allows prodrug conversion to cisplatin in the reducing environment of the tumor tissue	[69]
Supramolecular complex composed of FA-M-β-CyD and adamantane-grafted hyaluronic acid	-	Greater cytotoxic effect on colon cancer HCT-116 cells than FA-M-β-CyD alone Efficient cellular internalization, resulting in mitophagy-mediated cell death Antiproliferative potential	[70]
Folate-targeted PEG-modified amphiphilic cyclodextrin nanoparticles	Rg3 and quercetin	Prolonged blood circulation Enhanced tumor targeting in a colorectal cancer mouse model Lengthened animals survival in combination with anti-PD-L1	[71]
Amphiphilic cationic cyclodextrin nanoparticles modified with PEGylated folate	Docetaxel and siRNA	Significantly retarded tumoral growth Enhanced apoptotic activity of docetaxel with downregulation of RelA expression	[72]
Nanoparticles made of two different amphiphilic cyclodextrins coated with polyethylenimine or chitosan	Camptothecin	Greater cytotoxic effect on colon cancer HT29 cells than the free drug Enhanced Caco-2 cell permeability Significantly higher mucosal penetration than the free drug	[73]
Channel-type nanoparticles made of mannose-modified γ-cyclodextrin	Regorafenib	Attenuates inflammation and inhibits TAM activation Suppresses tumor cell proliferation and lesion neovascularization, and remodels the TME	[74]
Acrylic/maleic copolymer combined with β-cyclodextrin	Capecitabine	pH-responsive delivery system Targeted and controlled drug release	[75]
Alginate-based hydrogel crosslinked with modified β-cyclodextrin	5-Fluorouracil	Greater cytotoxic effect on colon cancer HT-29 cells than the free drug High and rapid accumulation in tumor cells, resulting in apoptosis	[76]
PBA-(ZW)-CD	Doxorubicin and ulixertinib	Enhanced tumor accumulation compared to free drugs Improved antitumor efficacy in heterotopic and orthotopic colorectal cancer models Tumor penetration comparable to free drugs	[77]
β-Cyclodextrin loaded in chitosan particles	Tetrahydrocurcumin	Immediate cellular uptake in colon cancer Caco-2 cells Displayed a dose-dependent cytotoxic activity	[78]
β-Cyclodextrin	Curcumin	Greater cytotoxic effect on SW480 and HCT-116 cells than free curcumin Decreased cancer cell viability, migration rates, and invasion rates Increased apoptosis rates by caspase 3 activation Improved water dispersibility	[79]
β-Cyclodextrin	Acetylshikonin	Greater cytotoxic effect on HCT-116 and MDA-MB-231 cells than the free therapeutic agent More pronounced cell cycle arrest and autophagy inhibition Increased accumulation of intracellular ROS	[80]

**Table 2:** Overview of chitosan-based drug delivery systems for colorectal cancer.

Drug Delivery System	Carried Agent(s)	Main Observations	Ref.
Folate-decorated lipid chitosan hybrid nanoparticles	5-Fluorouracil	Greater cytotoxic effect on colon cancer HT-29 and HCT-116 cells than the free drug Enhanced cellular uptake Biphasic release pattern: initial burst release followed by a sustained release for 48 h	[81]
Folic acid-conjugated chitosan/chondroitin sulfate self-assembled nanoparticles	Bortezomib	Enhanced cellular uptake and apoptosis in folate receptor-expressing colorectal cancer cells than in lung cancer cells pH-dependent release profile	[82]
Micelles made of amphiphilic chitosan modified with PEG and oleic acid	Camptothecin	Significant anticancer effects against HCT-116, Caco-2, and HT-29 cells Considerable reduction of tumor incidence and inflammation signs Safety profile for normal tissues	[83]
Chitosan-hyaluronic acid-protamine sulfate polyplexes	CRISPR/Cas9	Efficient gene delivery to HT-29 cells Downregulated ERCC1 and restored drug sensitivity in oxaliplatin-resistant cells	[84]
Carboxymethyl dextran–chitosan nanoparticles	Doxorubicin and siRNA	Induced apoptosis and inhibited migration of HCT-116 cells Significantly modified EMT gene expression	[32]
Chitosan quaternary ammonium salt	Bufadienolide nanocrystals	Effective targeting of intestinal sites Antitumor activity through Caspase-3 and Bax/Bcl-2 ratio pathways Significant apoptosis induction Enhanced ROS generation	[85]
Mesoporous silica nanoparticles capped with chitosan–glucuronic acid	Capecitabine	Higher uptake in HCT-116 cells Reduction in tumors, aberrant crypt foci, dysplasia, and inflammation Alleviation of toxic features	[86]
Poly(3-hydroxybutyrate)/chitosan-graft poly (acrylic acid) conjugated with sodium hyaluronate	Methotrexate	Greater cytotoxic effect on colon cancer Caco-2 cells than the free drug Enhanced ROS generation Increased apoptosis rates and elevated levels of DNA breakage inside tumor cells	[87]
Nanogels made of chitosan and carboxymethyl chitosan	Doxorubicin	Effective cellular internalization in colorectal cancer cells Prolonged contact time of the formulation onto the intestinal mucosa and an improved local drug concentration	[88]
Chitosan–carrageenan nanoparticles	Imatinib mesylate-poly sarcosine	Great potential for active targeting Promising for reducing the dose-dependent toxicity of carried freight	[89]
Chitosan–alginate nanoparticles	Curcumin diethyl diglutarate	Significantly enhanced stability, digestibility, bioaccessibility, and cellular uptake in Caco-2 cells	[90]
Chitosan–thiolated pectin composite	5-Fluorouracil	Targeted cytotoxicity towards HT-29 colorectal cells with milder cytotoxicity towards normal HEK-293 cells	[91]
Chitosan-coated magnetic cellulose nanowhiskers	5-Fluorouracil	Appropriate physicochemical properties to ensure a high tumor-penetrating capacity pH-dependent swelling and drug release performance Potent killing effects against colorectal cancer cells	[92]
Superparamagnetic chitosan-based nanocomplexes	Poly(L-glutamic acid)-SN-38 prodrug	Significant enhancement of tumor-targeted accumulation and cellular uptake Superior targeting and antitumor efficacy in colorectal cancer model mice	[93]
Chitosan-based polyelectrolyte complexes embedded with superparamagnetic nanoparticles	Irinotecan	Greater anti-colon cancer cell efficacy than the free drug Effective internalization by colon tumor cells Favorable tumor-targeting ability under the guidance of a magnetic field	[94]

**Table 3:** Overview of cyclodextrin-based drug delivery systems for liver cancer.

Drug Delivery System	Carried Agent(s)	Main Observations	Ref.
β-Cyclodextrin	Benzimidazole	pH-sensitive drug release with efficient uptake by HepG2 cells Inhibited liver cell proliferation through apoptosis induction	[98]
Cyclodextrin derivative (R6H4-CMβCD)-based nanoparticles	Curcumin	pH-sensitive drug release with efficient uptake by hepatoma cells High apoptosis rates in targeted cells with excellent anticancer effects	[99]
β-Cyclodextrin-gated mesoporous nanoparticles functionalized with an azobenzene/galactose-grafted polymer	Doxorubicin	Redox-sensitive drug release accelerated under UV irradiation Enhanced cytotoxicity to HepG2 cells compared to HeLa and COS7 cells	[100]
Folic acid–polyethylene glycol–β-cyclodextrin nanoparticles	Doxorubicin	Targeted and controlled medicine release to HepG2 cells Good encapsulation efficiency, blood compatibility, enhanced drug solubility	[101]
Folate-targeted polyethylene glycol-modified amphiphilic cyclodextrin nanoparticles	Melarsoprol	Achieved cell-specific uptake, cytotoxicity, apoptosis, and migration inhibition in targeted cells Prolonged the survival of mice with orthotopic tumors without causing side toxicity	[102]
Cross-linked γ- and β-cyclodextrin polymers	Doxorubicin and oxaliplatin	Greater cytotoxic effect on cancer cells than the free drugs Higher accumulation of the chemotherapeutic inside the cells	[103]
Glycyrrhetinic acid–β-cyclodextrin-grafted pullulan nanoparticles	Doxorubicin	High cellular uptake, with significant drug accumulation in the liver and a decreased concentration in the heart and kidneys Slow drug release Better therapeutic outcomes than the free drug	[104]
Biotinylated β-cyclodextrin-grafted pullulan	Doxorubicin	High cellular uptake, with significant drug accumulation in the liver and a decreased concentration in the heart and kidneys Sustained drug release Inhibited tumor cell growth	[105]
GSH-responsive cyclodextrin-based nanosponges	Doxorubicin	Comparable cytotoxicity and hepatic accumulation to the free drug Contribute to overcoming drug resistance by being taken up by tumor cells through an active mechanism and escaping the efflux drug pump	[106]
Magnetite–graphene oxide coated with a β-cyclodextrin–cholic acid–hyaluronic acid polymer	Camptothecin	Strong antitumor effect Induced local hyperthermia that produced tumor cell apoptosis	[107]
Polycationic amphiphilic β-cyclodextrin nanoparticles	-	Induced apoptosis and the lowered cell proliferation rate of HepG2 cells Hindered multidrug resistance	[108]

**Table 4:** Overview of chitosan-based drug delivery systems for liver cancer.

Drug Delivery System	Carried Agent(s)	Main Observations	Ref.
Chitosan and folic acid-functionalized chitosan nanoparticles	Doxorubicin	Inhibited tumor cell growth by promoting apoptosis and arresting cell cycle at G2/M phase through the p53/PRC1 pathway	[109]
Multifunctional thiolated chitosan derivatives	Arsenic trioxide	Glutathione-sensitive drug release Significantly improved tumor intracellular accumulation of the carried drug High tumor inhibition rate in mice with liver cancer	[110]
Micelles based on poly-ε-caprolactone linked to carboxymethyl chitosan through a disulfide bond and functionalized with glycyrrhetinic acid	Doxorubicin and pheophorbide A	Redox-responsive drug release Enhanced intracellular uptake by HepG2 cells Synergistic activity with the carried drugs Enhanced near-infrared imaging performance	[111]
Galactosylated chitosan-modified nanoparticles based on PEG-PLGA	Curcumin	Effectively internalized by HepG2 cells Greater inhibition of tumor growth than free curcumin	[112]
Chitosan/alginate nanoparticles	Curcumin diglutaric acid	Slow cumulative release of the carried agent in simulated gastrointestinal fluids without enzymes and in body fluid Better anticancer activity against Caco-2, HepG2, and MDA-MB-231 cells compared to the free drug	[113]
Chitosan-coated solid lipid nanoparticles	Zedoary turmeric oil	Significantly improved bioavailability Higher liver accumulation than uncoated particles Chitosan coating enhanced the internalization of particles by cells due to charge attraction	[114]
Micelles made of deoxycholic acid–O-carboxymethyl chitosan and A54 peptide	Ginsenoside compound K	pH-responsive and sustained drug release Greater cytotoxic effect on colon cancer HepG2 and Huh-7 cells than the free drug Promoted protein expression levels of caspase-3, caspase-9, and poly (ADP-ribose) polymerase	[115]
Erythrocytes loaded with chitosan nanogels	Pravastatin	Sustained drug release over 48 h Reduced the cellular viability of HepG2 cells	[116]

**Table 5:** Overview of cyclodextrin and chitosan-based drug delivery systems for gastric cancer.

Drug Delivery System	Carried Agent(s)	Main Observations	Ref.
Cyclodextrin-based polymer nanoparticles	Camptothecin	High in vitro cytotoxicity and strong antitumor activity in vivo Considerably decreased carbonic anhydrase, VEGF, and CD31 protein expression	[119]
Cholesterol-loaded chitosan nanoparticles	Salinomycin and siRNA	Superior in vitro cytotoxicity against two gastric carcinoma cells (i.e., SNU-668 and SGC-791) No significant adverse effects	[117]
Chitosan–hydrocaffeic acid conjugate gastric patch	Regorafenib	Sustained drug release for 8 days after oral administration Significant reduction in the tumor volume over 7 days	[120]
Chitosan oligosaccharide	Selenium	Effectively elevated phagocytosis and increased the secretion of anti-inflammatory cytokines in mouse peritoneal macrophages Possessed a significant immuno-enhancing effect with no cytotoxicity	[121]
N-deoxycholic acid–glycol chitosan functionalized with GX1–PEG–deoxycholic acid	Docetaxel	Sustained drug release accelerated by an acidic pH	[122]
Carboxymethyl chitosan	Norcantharidin	Upregulated the expression of TNF-α and Bax Downregulated the expression of VEGF, Bcl-2, MMP-2, and MMP-9 Enhanced antitumor efficacy against SGC-7901 cells, inhibiting tumor metastasis and inducing apoptosis in vivo	[123]
Chitosan-modified amino-magnetic nanoparticles	Copper oxide nanoparticles	Very low cell viability of human gastric and colorectal carcinoma cell lines	[124]
Superporous hydrogels made of chitosan–PVA blends	Resveratrol solid dispersion	Efficient drug release, sustained over 12 h Exhibited anti-inflammatory activity	[125]
α-, ß-, and γ-Cyclodextrins	New Zealand propolis	Inhibited the proliferation of four human gastro-intestinal cancer cell lines (i.e., DLD-1, HCT-116, NCI-N87, and KYSE-30) Strongly anti-inflammatory in vitro Strong lipid antioxidant activity	[126]

**Table 6:** Overview of cyclodextrin-based drug delivery systems for pancreatic cancer.

Drug Delivery System	Carried Agent(s)	Main Observations	Ref.
Cyclodextrin	α-Bisabolol	Considerable changes in the cytomorphology of pancreatic tumor cells Reduced tumor volume and lower Ki67-positive cells Induced tumor cell apoptosis and suppressed the phosphorylation of focal adhesion kinase	[130]
Hydroxy-propyl-β-cyclodextrin	PTA34 and PTA73	High antitumor activity towards PDAC cells Strong G2/M phase arrest followed by the induction of apoptosis	[131]
Hydroxy-propyl-β-cyclodextrin	Difluorinated curcumin	Inhibited colony and spheroid formation Induced cell cycle and apoptosis in PDAC cell lines	[132]
Multisubstituted-PEGylated β-cyclodextrins	Adamantane-modified bromelain	High antitumor activity due to long blood retention and increased tumor accumulation Enhancer of the anticancer effects of conventional chemotherapeutics	[128]
β-Cyclodextrin nanosponges	Erlotinib	Higher intracellular uptake and cytotoxicity in MIA PaCa-2 and PANC-1 cells compared to the free drug Reduced dose-related side effects	[133]

**Table 7:** Overview of chitosan-based drug delivery systems for pancreatic cancer.

Drug Delivery System	Carried Agent(s)	Main Observations	Ref.
Chitosan nanoparticles	Quercetin and 5-fluorouracil	Significant toxicity towards MIA PaCa2 pancreatic cancer cells	[134]
Folate-functionalized chitosan nanoparticles	Gemcitabine	Better absorption rate than nonfunctionalized carriers Preferential accumulation in human pancreatic cancer xenografts Significant inhibition of COLO357 cell proliferation	[135]
Chitosan oligosaccharide	Celastrol	Significantly inhibited tumor growth, induced apoptosis, and suppressed tumor metastasis of pancreatic cancer Lowered hepatic cytotoxicity	[136]
Liposomal nanoparticles coated with chitosan–folate	Lawsone	Strong free radical scavenging properties Significant inhibition of pancreatic cancer cell proliferation Increased cellular uptake Upregulated the Caspase 3, 9, and Bax genes	[137]
Chitosan-coated solid lipid nanoparticles	Ferulic acid and aspirin	Significantly reduced cell viability in MIA PaCa-2 and Panc-1 cells	[138]
